# Bridging urban-rural disparities in malaria care during pregnancy in Senegal: evidence from household and health facility surveys

**DOI:** 10.1186/s40249-025-01341-5

**Published:** 2025-07-20

**Authors:** Yongsheng Jiang, Di Liang, Jinkou Zhao, Shailendra Prasad, Medoune Ndiop, Serigne Amdy Thiam, Ibrahima Diallo, Doudou Sene, Rose Mpembeni, Jiayan Huang

**Affiliations:** 1 School of Public Health, Global Health Institute, Fudan University, Shanghai, 200032 China; 2 Programmatic Monitoring Department, The Global Fund to Fight AIDS, Tuberculosis and Malaria, Geneva, 1218 Switzerland; 3 Center for Global Health and Social Responsibility, University of Minnesota, Minneapolis, 55455 Minnesota United States; 4 Head of Monitoring Evaluation at National Malaria Control Program (NMCP), Rue FN 20, Dakar, 25270 Senegal; 5 Research Officer at National Malaria Control Program (NMCP), Rue FN 20, Dakar, 25270 Senegal; 6 Deputy Coordinator at National Malaria Control Program (NMCP), Rue FN 20, Dakar, 25270 Senegal; 7 Coodinator at National Malaria Control Program (NMCP), Rue FN 20, Dakar, 25270 Senegal; 8 School of Public Health and Social Sciences, Muhimbili University of Health and Allied Sciences, Dar Es Salaam, 65001 Tanzania

**Keywords:** Intermittent preventive treatment, Pregnancy, Malaria service readiness, Urban-rural disparities, Senegal

## Abstract

**Background:**

Despite the World Health Organization’s recommendations, the uptake of Intermittent Preventive Treatment in pregnancy with sulfadoxine-pyrimethamine (IPTp-SP) in Senegal remains suboptimal, with disparities observed between urban and rural areas. More remains to be known about how malaria service readiness would affect the utilization of IPTp-SP.

**Methods:**

Data were obtained from seven annual rounds of Demographic and Health Surveys (DHS) and Service Provision Assessments (SPA) in Senegal from 2012 to 2019. Using sample domain linkage to link the databases at the regional level. A malaria service readiness index was calculated to quantify the malaria service delivery capacity within the service environment where women reside. The Heckman selection model was utilized to analyze the relationship between malaria service readiness and IPTp-SP utilization.

**Results:**

From 2012 to 2019, the average number of IPTp-SP doses received in Senegal was 1.66 (95% *CI: *1.65–1.68), higher in urban areas [1.73 (95% *CI: *1.71–1.75)] than rural areas [1.63 (95% *CI: *1.62–1.65)]. Each one-point increase in malaria service readiness led to a rise of 0.251 doses in IPTp-SP. The significant interaction (Coef. = − 0.523,* P* < 0.001) indicated that women in rural areas received fewer doses of IPTp-SP (0.089) than in urban areas (0.612) for every unit increase in malaria service readiness.

**Conclusions:**

Linking household and health facility surveys revealed significant room for improvement in malaria service readiness and IPTp-SP utilization in rural areas in Senegal. For better IPTp-SP coverage, differential strategies are required for urban and rural settings. Urban areas need to enhance malaria service readiness, while rural areas should focus on improving service readiness alongside infrastructure and community engagement to bridge the urban-rural disparities.

**Graphical abstract:**

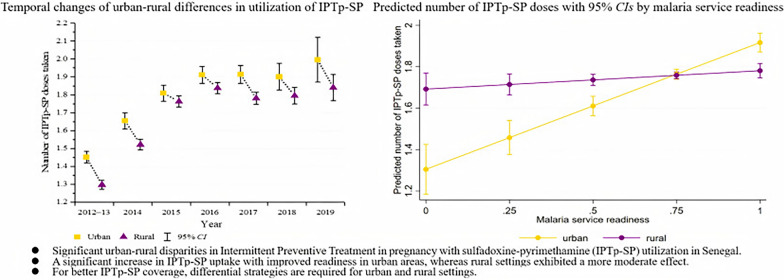

**Supplementary Information:**

The online version contains supplementary material available at 10.1186/s40249-025-01341-5.

## Background

Malaria in pregnancy (MiP) is a pervasive public health issue, posing significant risks to both mothers and newborns [1, 2]. In Africa, 34% of all 12.4 million pregnant women were exposed to malaria infection in 2023. Particularly, West Africa has the highest prevalence of MiP in Africa, at nearly 40% [1]. To mitigate the severe consequences of MiP, the World Health Organization (WHO) recommends that Intermittent Preventive Treatment in pregnancy with sulfadoxine-pyrimethamine (IPTp-SP) should be given at every scheduled antenatal care (ANC) beginning in the second trimester for all pregnant women as early as possible to ensure that women can receive at least three doses of SP (IPTp-SP3 +) [3].

Although most countries in sub-Saharan Africa (SSA) have incorporated IPTp-SP in their national malaria guidelines and have made notable progress, the coverage of IPTp-SP still falls short of the 80% target [1]. The underutilization of ANC services is among the major contributing factors to the gap [4]. Only 78% of pregnant women in SSA received at least one ANC visit. Moreover, even when pregnant women attend ANC, they might not receive IPTp-SP as recommended. Only 64% of pregnant women received at least one dose of SP (IPTp-SP1 +) in 2023 in SSA [1].

Previous studies have demonstrated that both individual-level and facility-level factors influence the uptake of IPTp-SP [5–9]. Individual factors, such as age, residence, education, and family economic status, are often unmodifiable. Institutional factors include inconsistent policy implementation, inadequate provider proficiency in IPTp-SP protocol, and drug stock-out. In recent years, service readiness, which refers to the ability and capacity of health facilities and providers to deliver services, has drawn attention in both research and policy practice [10]. The WHO comprehensively measures service readiness by trained staff, guidelines, equipment, diagnostic capacity, and medicines [10]. Evidence has suggested that improving malaria service readiness within health facilities can enhance the accessibility of malaria services, including IPTp-SP [11, 12]. However, limited by data and methodological constraints, these studies have primarily focused on IPTp-SP uptake at facility level, which may not accurately reflect actual IPTp-SP utilization among women at national or regional level. In addition, prior research has highlighted disparities in IPTp-SP uptake between urban and rural areas. A study conducted in Côte d'Ivoire revealed a lower IPTp-SP utilization rate in urban areas, attributed to inadequate health awareness [13]. In contrast, studies from Tanzania, Malawi, and Nigeria reported that rural residents experienced lower uptake of IPTp-SP due to limited healthcare access, outdated medical information, insufficient SP inventory, and poverty [14–16].

Measuring the association between malaria service readiness and other institutional factors and the receipt of IPTp-SP during ANC can be challenging since these can only be observed in women who have attended ANC. However, these factors could also affect women’s ANC attendance through their confidence and trust in the health system [17]. Under such circumstances, the estimated effects might suffer from a sample selection bias that might occur when the women’s attendance at ANC visits is not random but driven by observable and unobservable variables [18].

This study assessed the relationship between malaria service readiness and IPTp-SP usage in Senegal. Senegal, a low to moderate malaria transmission country in West Africa, initiated IPTp-SP in 2005 as one of the first malaria interventions, with the administration of a first dose of IPTp-SP from the first trimester of pregnancy and a second dose of IPTp-SP a month later [19]. In 2015, the National Malaria Control Program (NMCP) in Senegal adopted the administration of several doses always spaced one month apart. The indicator for monitoring IPTp coverage from two doses of IPTp-SP to IPTp-SP3 + [20]. Since 2015, there has been a significant improvement in the use of IPTp-SP. The proportion of women taking IPTp-SP3 + has almost doubled from 11 to 20% [21]. However, IPTp-SP coverage in Senegal remains suboptimal. Approximately 25% of pregnant women in urban areas in Senegal received IPTp-SP3 + , compared with 17% in rural areas [21]. The disparities between urban and rural areas in Senegal, with rural areas adversely affected in the provision and utilization of IPTp-SP services, were likely due to differences in health resource allocation, training of professional personnel, infrastructure development, and scarcity of medical equipment [22, 23]. Senegal exemplifies the typical gap in IPTp-SP coverage but also offers a unique opportunity to investigate the relationship between malaria service readiness and IPTp-SP utilization with the continuous availability of data from both the individual-level Demographic and Health Surveys (DHS) and the facility-level Service Provision Assessment (SPA). This study sought to assess the role of malaria service readiness within service environment in affecting IPTp-SP usage and explore potential urban–rural disparities in Senegal.

## Methods

### Study design and data sources

We used a cross-sectional design and utilized secondary data extracted from annual DHS and SPA in Senegal from 2012 to 2019. DHS Program supports a range of data collection options that can be tailored to fit specific monitoring and evaluation needs of countries, such as DHS and SPA Surveys [24].

DHS are nationally representative household surveys that adopt a two-stage stratified cluster sampling design to collect samples. In the first stage, Enumeration Areas (EA) are obtained as primary sampling units by sampling with probability proportional to size from census files. In the second stage, households are selected within each EA representatively and the sample is stratified by urban and rural areas [24]. DHS collect data using four model questionnaires: a household questionnaire, a woman’s questionnaire, a man's questionnaire, and a biomarker questionnaire. Continuous DHS (SCDHS) has been conducted annually in Senegal since 2012. For the current analysis, we used the woman's questionnaire data to identify the background characteristics, ANC visits, and IPTp-SP utilization information for each woman. For this analysis, we focused on the last pregnancy of the pregnant woman during the survey period.

The SPA is a comprehensive health facility-based survey that aims to assess whether the availability and readiness of different health services in a country meet the WHO service readiness indicators by accessing health facility information on infrastructure, staffing, services provided, and readiness to provide care [10, 25]. The SPA surveys include four standard data collection tools: facility inventory, client observation protocols, exit interviews, and health provider interviews. Senegal began to conduct Continuous SPA (SCSPA) in 2012, collecting annually representative samples of institutions at both sub-national (regional) and national levels. Facility inventory interviews are conducted with the managers of sampled institutions. In addition, health service providers are randomly selected for interviews by establishment size and department type from those present on the survey day at each institution. Facility inventory and health provider interviews are conducted annually. For our analysis, we used data collected from facility inventory and health provider questionnaires to identify the malaria service readiness in each facility.

### Linkage between DHS and SPA, and study sample

We linked data collected from the DHS woman’s questionnaires with data collected from the SPA facility and health provider questionnaires during the 2012–2019 survey rounds to provide greater insight into how malaria service readiness in service environment near women’s homes might influence their IPTp-SP usage. In Senegal, the DHS and SPA surveys determine regions and urban/rural areas based on national administrative boundaries, with the same sample domains used for sampling. Survey sample domains are usually the first administrative boundary level (i.e. region, province) and can be further defined by urban/rural areas within each region. The SCSPA constitutes a sampling investigation that does not have access to precise data from all health facilities for database linkage; nonetheless, it maintains representativeness at the regional (sample domain) level. Additionally, throughout the period from 2012 to 2019, both DHS and SPA surveys in Senegal have the same geographic regions as part of their sampling domains in each survey round. Sample domain linkage is a method to connect DHS and SPA data by linking them at the sample domain level, suitable when both surveys have the same geographic regions as part of their sampling domains. To make links more appropriate, we used the sample domain linkage proposed by the DHS Program to link the database [26].

**Fig. 1:**
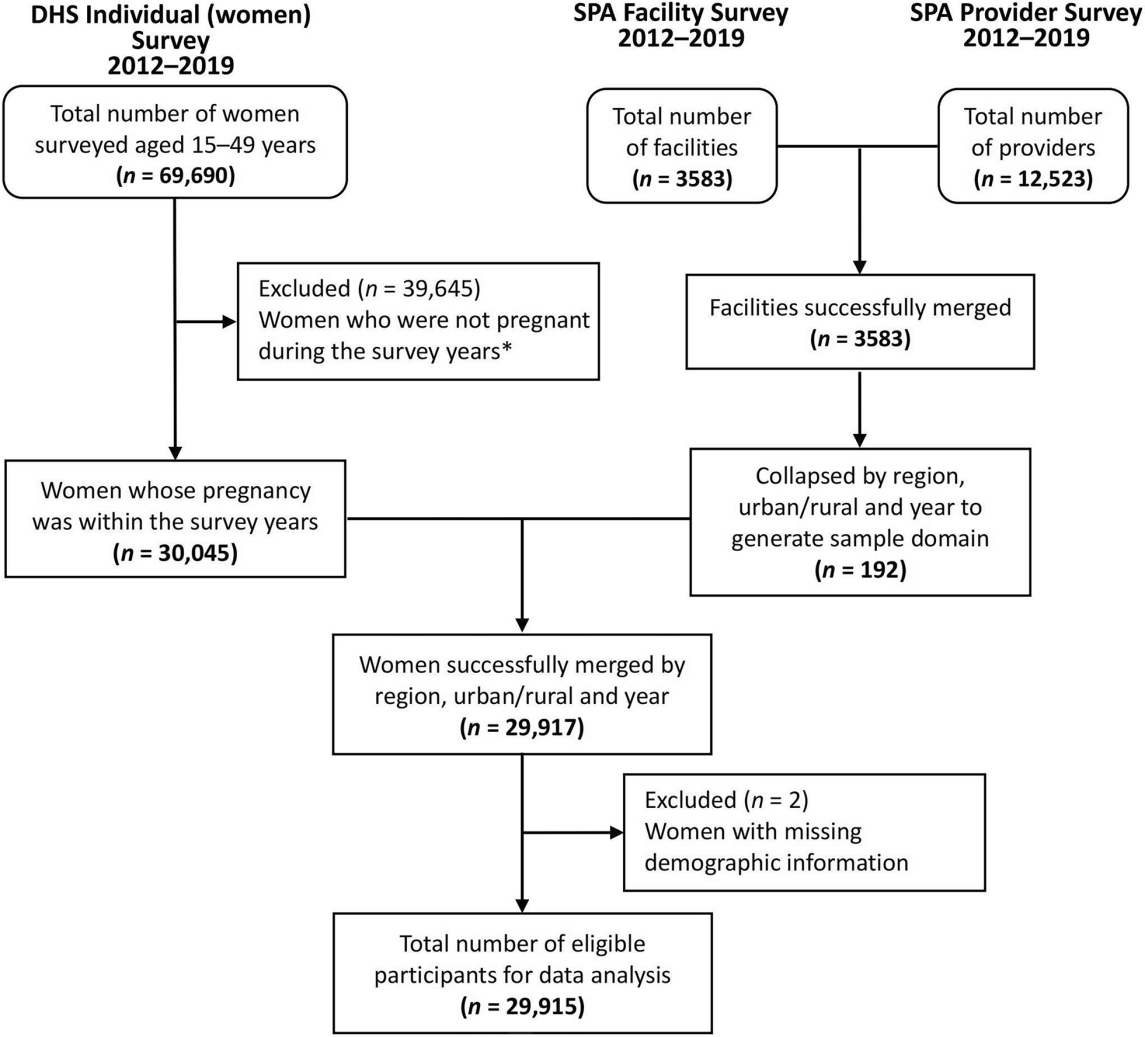
Flowchart of sampled individuals from DHS and SPA surveys conducted from 2012 to 2019. *Filtering criteria: last birth year ≥ 2012, if last birth year = 2012: last birth month ≥ 7 (ensuring that the IPTp-SP utilization for all women was within or after the year 2012, aligning with the survey years of the DHS and SPA)

We used a three-step process to link health facility data from SPA to DHS household survey data. First, we longitudinally combined the data of the DHS woman's questionnaire from 2012 to 2019 to screen women whose pregnancies occurred during the survey period, and a total of 30,045 women were obtained. The filtering criteria specified that the woman's last pregnancy year must be 2012 or later. For those whose last pregnancy year was exactly 2012, their last pregnancy month must be July or later, ensuring that the IPTp-SP utilization for all women was within or after the year 2012, aligning with the survey years of the DHS and SPA. Second, data from the SPA facility and health provider questionnaires from 2012 to 2019 were merged longitudinally and matched according to institution identification number, and then collapsed by three indicators of year, region, and urban/rural to obtain a total of 192 sample domains. Third, the screened DHS data (30,045 women) were matched with sample domains from SPA data by year, region, and urban/rural, resulting in a linked dataset of 29,917 pregnant women. Only women with complete information for characteristics were included in this study (excluding two pregnant women with missing demographic information), and the final sample size was 29,915. The inclusion and exclusion criteria used to link surveys and determine the eligible participants were outlined in Fig. 1.

### Outcome variable

Our outcome variable in this study is the number of IPTp-SP received by pregnant women during ANC in the last pregnancy. The measurement involved two questions from the DHS woman's questionnaire, “Did you consult for prenatal care during this pregnancy?” and “How many times did you take SP during pregnancy?”. The number of IPTp-SP received by pregnant women who attended ANC was selected as the outcome variable in this study, while the outcome variable of those who did not attend ANC was coded as missing values.

### The primary predictor of interest

The primary predictor variable is the malaria service readiness in each sample domain. In line with the WHO guideline and previous studies, this study computed a malaria service readiness index to denote the mean level of malaria service readiness in each sample domain [10, 27, 28]. The readiness index was calculated using four dimensions, each of which included several tracer items (Table 1). We determined a percentage score for each item and then calculated the mean of these to give an overall malaria service readiness index for each sample domain.

**Table 1:** Definitions of malaria service readiness

Dimension	Tracer item(s)	Definition of tracer	Percentage score^a^
Guidelines	Guidelines for diagnosis and treatment of malaria	Country-specific guidelines observed in service area	Proportion of facilities that have national guidelines for diagnosis and treatment of malaria among facilities offering malaria diagnosis/treatment services
	Guidelines for IPT	Country-specific IPT guidelines observed in service area	Proportion of facilities that have guidelines for IPT among facilities offering malaria diagnosis/treatment services
Trained staff	Staff trained in malaria diagnosis and treatment	At least one staff member providing the service trained in some aspect of malaria diagnosis and treatment in the last two years	Proportion of facilities with personnel trained in malaria diagnosis and treatment among facilities offering malaria diagnosis/treatment services
	Staff trained in IPT	At least one staff member providing the service trained in some aspect of IPT in the last two years	Proportion of facilities with personnel trained in IPT among facilities offering malaria diagnosis/treatment services
Diagnostics	Malaria diagnostic capacity	Facility offers any malaria test (laboratory or rapid test)	Proportion of facilities that have malaria diagnostic capacity among facilities offering malaria diagnosis/treatment services
Medicines and commodities	First-line antimalarial in stock	Artemisinin-based combination therapy (ACT) Infant	Proportion of facilities with ACT for infant among facilities offering malaria diagnosis/treatment services
		ACT Adolescent	Proportion of facilities with ACT for adolescent among facilities offering malaria diagnosis/treatment services
		ACT Adult	Proportion of facilities with ACT for adult among facilities offering malaria diagnosis/treatment services
	IPT drug	Sulfadoxine + Pyrimethamine (SP)	Proportion of facilities with SP among facilities offering malaria diagnosis/treatment services

*IPT* Intermittent preventive treatment

^a^sample weights are considered when calculating the Percentage score

### Covariates

Consistent with prior research, to control for confounding, the regression model included individual-level covariates, encompassing age (categorized in 15–19, 20–24, 25–29, 30–34, 35–39, 40–49 age groups), education level (none, primary, and secondary and higher), household wealth-index (poorest, poorer, middle, richer, and richest), religious type (Muslim, and others), place of residence (urban or rural) and the number of children aged 5 and under in household [5–9]. The household wealth index was generated using principal component analysis, which considers various factors such as the ownership of household amenities, the availability of a source of drinking water, and the type of flooring material used in the main house [29].

### Data analysis

We used descriptive analysis to describe the characteristics of women. Boxplots grouped by urban and rural areas were used to analyze the spatial-temporal changes of urban-rural differences in malaria service readiness and the utilization of IPTp-SP. Categorical variables were expressed as frequency (n) and proportion (%); continuous variables were reported as mean ± standard deviation (SD).

According to our definition of the outcome variable, only women who attended ANC visits could observe their IPTp-SP uptake, with missing values in other cases. In this context, missing values in the outcome variable were generated non-randomly, and ignoring missing values might lead to sample selection bias. To address this sample selection bias, the Heckman selection model was used to estimate the determinants of IPTp-SP utilization with two specifications [30]. The first specification used malaria service readiness as the main explanatory variable, adjusting for women’s characteristics. The second specification added the interaction between urban-rural settings and malaria service readiness.

For the Heckman selection model, a two-equation model, we estimate in the first step the following probit model for the probability of ANC visits for the full sample:

1 $$\text{Pr}\left({Z}_{i}=1\right)=\Phi ({\gamma W}_{i}+{\nu }_{i})$$

where the dependent variable, ${Z}_{i}$, is a binary variable defined as “whether a woman attended ANC visits in the last pregnancy” and dichotomized as “0” = “No ANC” or “1” = “at least one ANC visit in the last pregnancy”. $\Phi (.)$ is the cumulative distribution function of the standard normal distribution. ${W}_{i}$ are a series of variables that may affect ${Z}_{i}$, including readiness index, religion, age, number of children 5 and under in household, education level, household wealth-index, year, and type of place of residence. $\gamma$ denotes the parameter to be estimated and ${\nu }_{i}$ is an error term.

We calculate the non-selection hazard—the Inverse Mills Ratio (IMR) as defined by Heckman—from the estimated parameters of the selection Eq. (1) of women’s attendance of ANC:

2 $${\text{IMR}}=\frac{\phi ({\gamma W}_{i}+{\nu }_{i})}{\Phi ({\gamma W}_{i}+{\nu }_{i})}$$

where $\phi (.)$ is the probability density function.

For the second model, we used Ordinary Least Squares (OLS) regression with robust standard errors, included pregnant women participating in ANC visits, and incorporated IMR into the equation, as follows:

3 $${y}_{i}=\beta {x}_{i}+{\lambda {\text{IMR}}}_{i}+{\mu }_{i}$$

where the dependent variable, ${y}_{i}$, denotes the number of IPTp women received during ANC visits. ${x}_{i}$ are a series of independent variables that may affect ${y}_{i}$. The key independent variable is the readiness of malaria services. Other control variables are residence, education level, religion, age, and household wealth-index. $\beta$ represents the parameter to be estimated, *λ* is the estimator of IMR, and ${\mu }_{i}$ is the error term of a normal distribution with a mean of zero and a standard deviation $\upsigma$ to be estimated. If *λ* is tested to be significantly non-zero, sample selection bias exists; otherwise, sample selection bias does not exist. A negative *λ* implies that unobserved factors increasing ANC attendance are associated with lower IPTp-SP doses. This would lead ordinary OLS estimates to overstate the effect of malaria service readiness. A positive *λ* implies that unobserved factors increasing ANC attendance are linked to higher IPTp-SP doses, causing ordinary OLS to understate the true effect.

The Skewness-Kurtosis Test and Q-Q plot (Additional file 1: Figure S1) were used to assess the normality assumption of OLS residuals. Residual diagnostics indicated approximate normality, with skewness (0.04) and kurtosis (2.61) within acceptable ranges for normality.

This study conducted a sensitivity analysis to compare the correlation of malaria service readiness on IPTp-SP utilization between women with only one pregnancy and those with multiple pregnancies, and employed the suest (seemingly unrelated regression) test in Stata version 17.0 (Stata Corp, College Station, USA). The robustness analysis was conducted to assess the stability of the model by excluding samples from both the youngest age group (15 to 19 years) and the oldest age group (40 to 49 years). All the analyses were conducted using Stata 17 [31].

## Results

### Characteristics of urban and rural pregnant women

Among 29,915 pregnant women, the mean age was 29.6 (SD = 7.4) years, with a range of 15–49 years. Almost 70% (20,358, 68.1%) of them were living in rural areas and 97% (29,030, 97.0%) were Muslim. In rural areas, the proportion of pregnant women with no education reached about 75% (15,117, 74.3%). About 74% of pregnant women in rural areas lived in the poorer (8496, 41.7%) or poorest (6599, 32.4%) wealth quintile, compared with less than 17% (1632, 17.1%) in urban areas (Table 2).

**Table 2:** Characteristics of pregnant women among urban and rural areas in Senegal from 2012 to 2019

	Total (*n* = 29,915) *n* (%)	Urban (*n* = 9557) *n* (%)	Rural (*n* = 20,358) *n* (%)
**Highest educational level**			
No education	19,540 (65.3)	4423 (46.3)	15,117 (74.3)
Primary	6026 (20.1)	2710 (28.4)	3316 (16.3)
Secondary and higher	4349 (14.5)	2424 (25.4)	1925 (9.5)
**Current marital status**			
Married	27,869 (93.2)	8542 (89.4)	19,327 (94.9)
Others	2046 (6.8)	1015 (10.6)	1031 (5.1)
**Religion**			
Muslim	29,030 (97.0)	9214 (96.4)	19,816 (97.3)
Others	885 (3.0)	343 (3.6)	542 (2.7)
**Age(years)**			
15–19	2111 (7.1)	495 (5.2)	1616 (7.9)
20–24	6026 (20.1)	1766 (18.5)	4260 (20.9)
25–29	7464 (25.0)	2467 (25.8)	4997 (24.6)
30–34	6408 (21.4)	2256 (23.6)	4152 (20.4)
35–39	4472 (15.0)	1498 (15.7)	2974 (14.6)
40–49	3434 (11.5)	1075 (11.3)	2359 (11.6)
**Mean age (years), mean (± SD)**	29.6 ± 7.4	30.0 ± 7.1	29.4 ± 7.5
**Wealth index quintile**			
Poorest	8982 (30.0)	486 (5.1)	8496 (41.7)
Poorer	7745 (25.9)	1146 (12.0)	6599 (32.4)
Middle	6240 (20.9)	2676 (28.0)	3564 (17.5)
Richer	4227 (14.1)	3020 (31.6)	1207 (5.9)
Richest	2721 (9.1)	2229 (23.3)	492 (2.4)
**Number of children 5 and under in household**			
0	770 (2.6)	351 (3.7)	419 (2.1)
1	5230 (17.5)	2378 (24.9)	2852 (14.0)
2–3	12,585 (42.1)	4183 (43.8)	8402 (41.3)
≥ 4	11,331 (37.9)	2645 (27.7)	8685 (42.7)

Pregnant women sample pools by the linkage of DHS and SPA Surveys in Senegal from 2012 to 2019. *n* stands for number. Results were unweighted

*SD* Standard deviation

### Spatial-temporal changes of urban–rural differences in malaria service readiness and IPTp-SP utilization

**Fig. 2:**
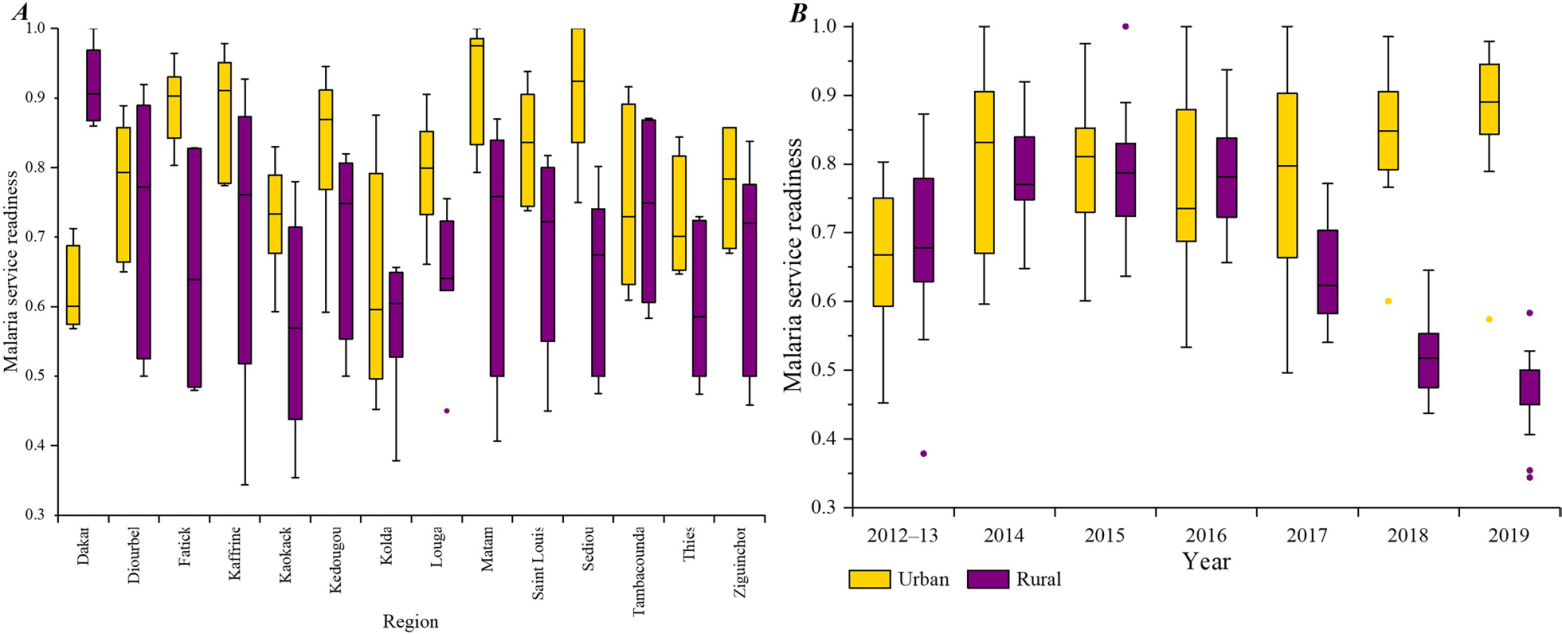
Spatial-temporal Changes of Urban–rural Differences in Malaria Service Readiness. **A** depicts the median and interquartile ranges for malaria service readiness between rural and urban health facilities for each region in Senegal from 2012 to 2019. **B** shows the median and interquartile ranges for malaria service readiness between rural and urban health facilities in Senegal by year. The x-axis on the left is the region in Senegal, and the x-axis is the year on the right. The y-axis is the malaria service readiness. In each boxplot, the horizontal line represents the median service readiness in rural or urban facilities, the box indicates the first and third quartiles, the whiskers extend the observed value closest to 1.5 times the interquartile range below the 25th percentile or above the 75th percentile, and the points represent observed values outside the interval. The gold and purple in the boxplots represent urban and rural areas respectively

In most regions, the median values of malaria service readiness in rural areas were lower than in urban areas. The median value in urban areas generally increased year by year since 2012, while in rural areas it increased from 2012 to 2015, peaked at 0.79 in 2015, and then decreased year by year till 2019. The gap between urban and rural areas widened in recent years, with the largest gap seen in 2019 (Fig. 2).

**Fig. 3:**
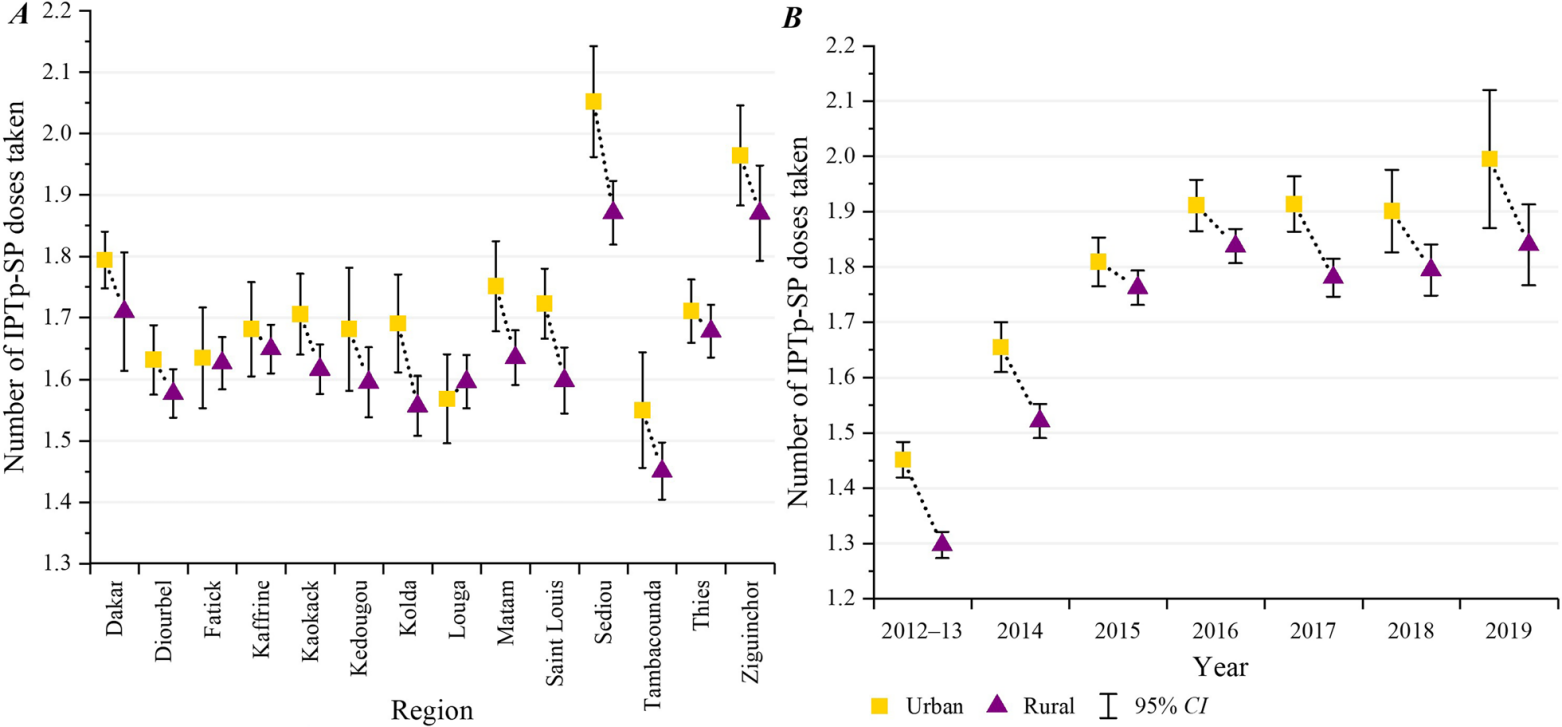
Spatial-temporal Changes of Urban–rural Differences in the Utilization of IPTp-SP. IPTp-SP: Intermittent preventive treatment of malaria in pregnancy with sulfadoxine-pyrimethamine. **A** depicts the mean and 95% confidence intervals of the number of IPTp-SP received by pregnant women during antenatal care visits between rural and urban health facilities for each region in Senegal from 2012 to 2019. **B** depicts the mean and 95% confidence intervals of the number of IPTp-SP received by pregnant women during antenatal care visits between rural and urban health facilities in Senegal by year. The x-axis on the left is the region in Senegal, and the x-axis is the year on the right. The y-axis is the number of IPTp-SP received by pregnant women during antenatal care visits. The gold squares and purple triangles in the figure represent the mean doses of the IPTp-SP for urban and rural areas, respectively. *CI* confidence interval

From 2012 to 2019, the average number of IPTp-SP in Senegal was 1.66 (95% *CI: *1.65–1.68), 1.73 (95% *CI: *1.71–1.75) in urban areas and 1.63 (95% *CI: *1.62–1.65) in rural areas. The number of IPTp-SP was higher in urban areas than in rural areas across most regions. Meanwhile, the number of IPTp-SP in urban and rural areas changed almost simultaneously from 2012 to 2019, with urban areas consistently higher than rural areas (Fig. 3).

### Malaria service readiness influencing IPTp-SP utilization in urban and rural areas

Table 3 presents the results of the Heckman selection model for malaria service readiness associated with IPTp-SP uptake. The model addressed the censored observations of 2126 women who did not attend ANC and the uncensored observations of 27,789 women who engaged in any form of ANC visits. Both specifications depicted satisfactory fit (Chi-square test: *P* < 0.001), which indicated that there was a selection effect of whether attending ANC on IPTp-SP usage. In addition, the *λ* values were significantly less than zero, indicating that after adjusting for sample selection bias, the positive influence of malaria service readiness on the uptake of IPTp-SP was modestly attenuated, but the association was still statistically significant.

**Table 3:** Determinants of the number of IPTp-SP in Senegal, as per Heckman selection model estimates

	(1)^1^	(2)^2^
Outcome equation for number of IPTp-SP (95% *CI*)		
**Readiness index**	0.251^***^ (0.162 to 0.340)	0.612^***^ (0.456 to 0.768)
**Place of residence**		
Urban^®^	1	1
Rural	− 0.002 (− 0.033 to 0.028)	0.387^***^ (0.245 to 0.529)
Readiness*Place of residence		
Readiness*Urban^®^	–	1
Readiness*Rural	–	− 0.523^***^ (− 0.709 to − 0.337)
**Education level**		
No education^®^	1	1
Primary	0.080^***^ (0.050 to 0.110)	0.081^***^ (0.051 to 0.111)
Secondary and higher	0.142^***^ (0.106 to 0.178)	0.141^***^ (0.105 to 0.177)
**Religion**		
Muslim^®^	1	1
Others	− 0.002 (− 0.070 to 0.066)	0.003 (− 0.065 to 0.070)
**Age (years)**		
15–19^®^	1	1
20–24	− 0.008 (− 0.058 to 0.041)	− 0.009 (− 0.059 to 0.040)
25–29	0.016 (− 0.032 to 0.064)	0.015 (− 0.033 to 0.064)
30–34	0.080^**^ (0.030 to 0.129)	0.078^**^ (0.029 to 0.128)
35–39	0.106^***^ (0.053 to 0.158)	0.106^***^ (0.053 to 0.158)
40–49	0.163^***^ (0.107 to 0.220)	0.165^***^ (0.109 to 0.221)
**Wealth index quintile**		
Poorest^®^	1	1
Poorer	0.021 (− 0.010 to 0.052)	0.020 (− 0.010 to 0.051)
Middle	0.026 (− 0.008 to 0.061)	0.028 (− 0.007 to 0.063)
Richer	0.047^*^ (0.004 to 0.089)	0.057^**^ (0.014 to 0.100)
Richest	0.036 (− 0.014 to 0.087)	0.054^*^ (0.003 to 0.105)
Selection Equation for ANC (95%* CI*)		
**Readiness index**	− 0.541^***^ (− 0.744 to − 0.338)	− 0.547^***^ (− 0.750 to − 0.343)
**Religion**		
Muslim^®^	1	1
Others	0.225^**^ (0.085 to 0.365)	0.221^**^ (0.081 to 0.361)
**Age (years)**		
15–19^®^	1	1
20–24	− 0.049 (− 0.164 to 0.065)	− 0.051 (− 0.165 to 0.064)
25–29	− 0.143^*^ (− 0.253 to − 0.033)	− 0.145^**^ (− 0.255 to − 0.035)
30–34	− 0.234^***^ (− 0.345 to − 0.123)	− 0.236^***^ (− 0.347 to − 0.125)
35–39	− 0.319^***^ (− 0.433 to − 0.206)	− 0.322^***^ (− 0.435 to − 0.208)
40–49	− 0.648^***^ (− 0.761 to − 0.536)	− 0.652^***^ (− 0.765 to − 0.539)
**Number of children aged 5 and under in household**		
0^®^	1	1
1	0.620^***^ (0.517 to 0.722)	0.621^***^ (0.518 to 0.724)
2–3	0.742^***^ (0.643 to 0.841)	0.744^***^ (0.644 to 0.843)
≥ 4	0.792^***^ (0.691 to 0.893)	0.793^***^ (0.691 to 0.894)
**Education level**		
No education^®^	1	1
Primary	0.119^***^ (0.055 to 0.182)	0.119^***^ (0.055 to 0.182)
Secondary and higher	0.168^***^ (0.084 to 0.252)	0.169^***^ (0.085 to 0.254)
**Wealth index quintile**		
Poorest^®^	1	1
Poorer	0.208^***^ (0.151 to 0.266)	0.209^***^ (0.151 to 0.266)
Middle	0.345^***^ (0.274 to 0.416)	0.345^***^ (0.274 to 0.416)
Richer	0.363^***^ (0.274 to 0.452)	0.365^***^ (0.276 to 0.454)
Richest	0.295^***^ (0.189 to 0.401)	0.296^***^ (0.190 to 0.402)
**Place of residence**		
Urban^®^	1	1
Rural	− 0.092^**^ (− 0.156 to − 0.028)	− 0.095^**^ (− 0.159 to − 0.031)
**Year**		
2012–2013^®^	1	1
2014	0.538^***^ (0.475 to 0.601)	0.538^***^ (0.476 to 0.601)
2015	0.955^***^ (0.881 to 1.029)	0.957^***^ (0.883 to 1.030)
2016	0.985^***^ (0.910 to 1.059)	0.989^***^ (0.914 to 1.064)
2017	0.956^***^ (0.874 to 1.038)	0.947^***^ (0.865 to 1.029)
2018	0.979^***^ (0.864 to 1.093)	0.957^***^ (0.842 to 1.072)
2019	0.977^***^ (0.797 to 1.156)	0.948^***^ (0.767 to 1.129)

*CI*: confidence interval; ^®^: Reference category; IPTp-SP: Intermittent preventive treatment of malaria in pregnancy with sulfadoxine-pyrimethamine; ANC: Antenatal care

^***^P < 0.001

^**^P < 0.01

^*^P < 0.05

^1^Specification 1 used the malaria service readiness as the main explanatory variable, adjusting for women’s characteristics. Number of observations (*n* = 29,915), Censored observations (*n* = 2126), Uncensored observations (*n* = 27,789), Wald chi2(14): 206.10, Prob > chi2: *P* < 0.001, and *λ*: − 0.75 (*P* < 0.001)

^2^Specification 2 added the interaction between urban–rural settings and the malaria service readiness. Number of observations (*n* = 29,915), Censored observations (*n* = 2126), Uncensored observations (*n* = 27,789), Wald chi2(15): 239.29, Prob > chi2: *P* < 0.001, and *λ*: − 0.75 (*P* < 0.001)

In Specification 1, each one-point increase in malaria service readiness was associated with an average increase of 0.251 doses of IPTp-SP, after controlling for confounding and adjusting for selection bias. After adding the interaction terms (Specification 2), malaria service readiness demonstrated a positive effect on IPTp-SP utilization. The result revealed a significant interaction between urban–rural settings and malaria service readiness (Coef. = − 0.523,* P* < 0.001). Specifically, for every unit increase in malaria service readiness, women in urban areas received an average increase of 0.612 doses of IPTp-SP, which is higher than the 0.089 doses observed in rural areas (Table 3 and Additional file 1: Figure S2).

The results of the sensitivity analysis showed that the suest test revealed no statistically significant difference (*P* = 0.909) in the correlation of malaria service readiness on IPTp-SP utilization between women with only one pregnancy and those with multiple pregnancies (Additional file 1: Table S1). The robustness analysis showed similar results to the main findings (Additional file 1: Table S2 and Figure S3).

## Discussion

This study uncovered notable urban–rural disparities in malaria service readiness and IPTp-SP utilization. The results suggested that an increase in malaria service readiness within the sample domain is significantly associated with an elevated uptake of IPTp-SP, with a pronounced effect observed in urban areas. Different from other studies conducted in Senegal to date [32–34], this study, for the first time, integrated data from the DHS and SPA databases, linking malaria service readiness in service environment with individual IPTp-SP utilization. Additionally, a Heckman selection model was utilized to address the issue of sample selection bias.

Association Between IPTp Utilization and Malaria Service Readiness. The average IPTp-SP doses received by pregnant women increased annually in both urban and rural areas; however, it remained below WHO and Senegal’s recommended minimum of three doses, consistent with trends in other malaria-endemic countries in SSA [6, 7, 14, 15]. Moreover, the trend of IPTp-SP3 + in Senegal was basically the same as that in most other SSA countries, which was relatively lagging behind the overall level of SSA [1]. This study found that the lack of malaria service readiness was a significant predictor of low IPTp-SP utilization, aligning with Bajaria's study in Tanzania, where high-readiness facilities showed increased IPTp-SP delivery [35]. When health facilities face barriers such as inadequate training of health providers and lack of SP stocks, the readiness of these facilities may deteriorate, potentially reducing the uptake of IPTp-SP [9, 34, 36–38]. For instance, SP stockouts were reported in many service delivery points in Senegal between 2017 and 2018 [39]. This shortage could have had a profound impact on the coverage and effectiveness of IPTp-SP.

However, unlike the previous study, our study expanded beyond health facilities to examine the broader service environment by calculating a readiness index that denoted the average level of malaria service readiness within a region. For each 0.1 unit improvement in malaria service readiness (equivalent to 10% more health facilities being ready for malaria service in a sample domain), pregnant women in that sample domain received an additional 0.025 doses of IPTp-SP on average. The Heckman selection model results further indicate that improved readiness not only exerts an influence on women who are already engaged with health facilities for ANC but also extends its impact to women within the community who had not previously sought services at these establishments. The enhanced readiness of health facilities may engender an increase in trust and a consequent rise in the willingness of women to engage with these services [17, 40].

### Urban–rural inequity in malaria service readiness and IPTp-SP utilization

IPTp-SP uptake is higher in urban areas than in rural areas, a trend observed in many other low- and middle-income countries in SSA [9, 14, 15]. An exception is noted in a study from Côte d'Ivoire, which reported greater IPTp-SP uptake in rural areas [13]. The discrepancy may be attributed to the unique context of the country and the time period of the study. The disparity in IPTp-SP uptake between urban and rural areas can be attributed to a major economic gap affecting demand [41], and supply-side challenges in health facility access and the training of the health workforce. Urban areas are typically endowed with superior infrastructure and medical resources [28, 42]. In contrast, rural areas often suffer from inadequate transportation infrastructure and a dearth of medical personnel, which hinders the delivery of proper training on IPTp-SP [43–45].

In recent years, malaria service readiness has seen a steady growth in urban areas in Senegal; in contrast, readiness in rural areas has been declining since 2016. This decline in rural readiness is significantly affected by the availability of essential drugs like SP and the implementation of updated guidelines [46]. Survey data from Senegal corroborated this, revealing instances of SP stockouts around 2017, with a more pronounced impact in rural regions where resources were already limited [39]. Low levels of SP stocks in rural health facilities in recent years, combined with possible delays in the spread of the latest malaria treatment protocols, may have contributed to the decrease in malaria service readiness.

The interaction analysis revealed a significant increase in IPTp-SP uptake in urban areas, driven by enhanced readiness, while rural settings exhibited a more moderate effect. This disparity could be attributed to the compounded challenges characteristic of rural environments, such as insufficient infrastructure, scarcity of educational resources, and economic hardships, which collectively impede both access to and utilization of malaria services [28, 47]. To further expand the coverage of IPTp-SP, urban areas demand an augmentation of malaria service readiness to amplify the scope of IPTp-SP. In rural areas, it's crucial to not only strengthen malaria service readiness but also focus on infrastructure development and community-level interventions to bridge the urban–rural disparities. Since 2021, the NMCP has undertaken a series of initiatives to improve malaria service readiness. Regarding personnel training, various training activities are organized to improve the diagnosis and treatment skills of health workers. These initiatives also focus on training community-level health workers for initial diagnosis and treatment. To bolster diagnostic capacity, a nationwide laboratory network has been established, and quality control systems have been implemented. In terms of drug supply, efforts will be made to ensure the continuous availability of SP. Additionally, the IPTp-SP strategy has been revitalized in highly rural districts to rebalance coverage between urban and rural areas, while also to shake up IPTp-SP coverage throughout the country [23].

## Limitations

This study has several limitations. First, although linking SPA with DHS women's data is useful to measure the relationship between malaria service readiness and maternal IPTp-SP service utilization, there are limitations to the sample domain linkage. The facility sampling utilized in SCSPA may lead to an underestimation of the adequacy of the health service environment. The SCSPA samples are statistically representative across large geographical areas (regions) within a country, rather than being designed to provide statistically representative estimates for smaller geographical areas, such as those surrounding a DHS cluster. Consequently, this high-level linkage may obscure potential variations within the sampling domains. In addition, the weakness of this study, which used a cross-sectional study design, is that we could not infer a causal relationship in time between IPTp-SP utilization and malaria service readiness.

## Conclusions

In conclusion, despite the NMCP’s IPTp-SP coverage improvement strategies of adopting three and more doses since 2015, the utilization of IPTp-SP in Senegal is suboptimal. This study highlights the importance of improving malaria service readiness and enhancing IPTp-SP utilization in Senegal, especially in rural areas. By linking health facility data with population data, we have established that the malaria service readiness in service environment exerts a significantly stronger positive correlation on IPTp-SP utilization in urban areas. Consequently, to optimize IPTp-SP utilization, it is crucial to sustain and enhance malaria service readiness in urban areas. Concurrently, rural areas should enhance service readiness while integrating targeted interventions from education, environmental health, and community engagement to mitigate urban–rural disparities.

## Supplementary Information

Additional file 1: Table S1. Determinants of the number of IPTp-SP in Senegal, as per Heckman selection model estimates (sensitivity analysis results). Table S2. Determinants of the number of IPTp-SP in Senegal, as per Heckman selection model estimates (robustness analysis results). Figure S1. Quantile-Quantile Plot of Residuals for Normality Assessment. Figure S2. Interaction plot of the effects of urban-rural settings and malaria service readiness on IPTp-SP. Figure S3. Interaction plot of the effects of urban-rural settings and malaria service readiness on IPTp-SP by robustness analysis results

## Data Availability

The datasets analyzed during the current study are available in the DHS Program, https://dhsprogram.com/.

## Abbreviations

ACT: Artemisinin-based combination therapy
ANC: Antenatal care
*CI*: Confidence interval
DHS: Demographic and Health Survey
EA: Enumeration areas
IPTp: Intermittent Preventive Treatment in pregnancy
IPTp-SP1 + /IPTp-SP3 +: Having had at least one/three doses of SP
MiP: Malaria in pregnancy
NMCP: National malaria control program
OLS: Ordinary least squares
SCDHS: Senegal continuous demographic and health survey
SCSPA: Senegal continuous service provision assessment
SD: Standard deviation
SP: Sulfadoxine-pyrimethamine
SPA: Service Provision Assessment
SSA: Sub-Saharan Africa
WHO: World Health Organization
