# Supplementary material for: Bridging urban-rural disparities in malaria care during pregnancy in Senegal: evidence from household and health facility surveys
Source: Infect Dis Poverty. 2025 Jul 20;14:71. doi: 10.1186/s40249-025-01341-5 (PMC12276681; doi:10.1186/s40249-025-01341-5)
Supplement: Supplementary file 1 — Additional file 1: Table S1. Determinants of the number of IPTp-SP in Senegal, as per Heckman selection model estimates (sensitivity analysis results). Table S2. Determinants of the number of IPTp-SP in Senegal, as per Heckman selection model estimates (robustness analysis results). Figure S1. Quantile-Quantile Plot of Residuals for Normality Assessment. Figure S2. Interaction plot of the effects of urban-rural settings and malaria service readiness on IPTp-SP. Figure S3. Interaction plot of the effects of urban-rural settings and malaria service readiness on IPTp-SP by robustness analysis results [file 40249_2025_1341_MOESM1_ESM.docx]

**Bridging urban-rural disparities in malaria care during pregnancy in Senegal: Evidence from household and health facility surveys**

**Table S1.** Determinants of the number of IPTp-SP in Senegal, as per Heckman selection model estimates (sensitivity analysis results)

|  | **(1)^1^** | **(2)^2^** |
| --- | --- | --- |
| **Outcome Equation for number of IPTp-SP (95% *CI*)** | | |
| **Readiness** **index** | 0.263^*^ (0.060 to 0.466) | 0.250^***^ (0.151 to 0.348) |
| **Place of residence** |  |  |
| Urban ® | 1 | 1 |
| Rural | -0.056 (-0.123 to 0.011) | 0.013 (-0.021 to 0.048) |
| **Education level** |  |  |
| No education ® | 1 | 1 |
| Primary | 0.027 (-0.040 to 0.094) | 0.089^***^ (0.055 to 0.122) |
| Secondary and higher | 0.069^*^ (0.007 to 0.132) | 0.150^***^ (0.104 to 0.197) |
| **Religion** |  |  |
| Muslim ® | 1 | 1 |
| Others | 0.008 (-0.126 to 0.143) | -0.011^*^ (-0.089 to 0.067) |
| **Age (years)** |  |  |
| 15–19 ® | 1 | 1 |
| 20–24 | 0.030 (-0.034 to 0.093) | -0.031 (-0.140 to 0.078) |
| 25–29 | 0.122^**^ (0.044 to 0.201) | 0.002 (-0.105 to 0.109) |
| 30–34 | 0.219^***^ (0.106 to 0.332) | 0.076 (-0.031 to 0.183) |
| 35–39 | 0.077 (-0.108 to 0.262) | 0.110^*^ (0.002 to 0.219) |
| 40–49 | 0.125 (-0.203 to 0.452) | 0.158^**^ (0.048 to 0.269) |
| **Wealth index quintile** |  |  |
| Poorest ® | 1 | 1 |
| Poorer | 0.068 (-0.006 to 0.142) | 0.009 (-0.025 to 0.043) |
| Middle | 0.029 (-0.052 to 0.110) | 0.023 (-0.015 to 0.062) |
| Richer | 0.017 (-0.079 to 0.113) | 0.051^*^ (0.004 to 0.099) |
| Richest | 0.003 (-0.105 to 0.112) | 0.036 (-0.022 to 0.094) |
| **Selection Equation for ANC (95% *CI*)** | | |
| **Readiness** **index** | -0.357 (-0.842 to 0.128) | -0.561^***^ (-0.787 to -0.336) |
| **Religion** |  |  |
| Muslim ® | 1 | 1 |
| Others | -0.051 (-0.309 to 0.208) | 0.315^***^ (0.150 to 0.481) |
| **Age (years)** |  |  |
| 15–19 ® | 1 | 1 |
| 20–24 | 0.017 (-0.123 to 0.158) | -0.007 (-0.250 to 0.236) |
| 25–29 | -0.222^**^ (-0.383 to -0.061) | -0.035 (-0.273 to 0.202) |
| 30–34 | -0.419^***^ (-0.624 to -0.214) | -0.126 (-0.363 to 0.111) |
| 35–39 | -0.276 (-0.599 to 0.048) | -0.233 (-0.471 to 0.005) |
| 40–49 | -0.608^*^ (-1.082 to -0.134) | -0.567^***^ (-0.805 to -0.329) |
| **Number of children aged 5 and under in household** | | |
| 0 ® | 1 | 1 |
| 1 | 0.407^***^ (0.207 to 0.608) | 0.692^***^ (0.571 to 0.814) |
| 2–3 | 0.463^***^ (0.268 to 0.658) | 0.857^***^ (0.740 to 0.975) |
| ≥ 4 | 0.452^***^ (0.251 to 0.652) | 0.916^***^ (0.797 to 1.035) |
| **Education level** |  |  |
| No education ® | 1 | 1 |
| Primary | 0.226^**^ (0.081 to 0.370) | 0.097^**^ (0.026 to 0.168) |
| Secondary and higher | 0.172^*^ (0.036 to 0.309) | 0.195^**^ (0.082 to 0.308) |
| **Wealth index quintile** |  |  |
| Poorest ® | 1 | 1 |
| Poorer | 0.169^*^ (0.023 to 0.315) | 0.218^***^ (0.155 to 0.282) |
| Middle | 0.410^***^ (0.229 to 0.590) | 0.345^***^ (0.267 to 0.424) |
| Richer | 0.328^**^ (0.132 to 0.524) | 0.398^***^ (0.297 to 0.499) |
| Richest | 0.399^**^ (0.167 to 0.631) | 0.298^***^ (0.176 to 0.420) |
| **Place of residence** |  |  |
| Urban ® | 1 | 1 |
| Rural | 0.053 (-0.089 to 0.195) | -0.128^***^ (-0.201 to -0.056) |
| **Year** |  |  |
| 2012–2013 ® | 1 | 1 |
| 2014 | 0.595^***^ (0.451 to 0.740) | 0.534^***^ (0.464 to 0.604) |
| 2015 | 1.073^***^ (0.893 to 1.253) | 0.933^***^ (0.852 to 1.015) |
| 2016 | 1.131^***^ (0.952 to 1.310) | 0.957^***^ (0.874 to 1.040) |
| 2017 | 1.172^***^ (0.972 to 1.371) | 0.911^***^ (0.820 to 1.001) |
| 2018 | 1.269^***^ (0.969 to 1.570) | 0.933^***^ (0.808 to 1.059) |
| 2019 | 1.331^***^ (0.882 to 1.780) | 0.904^***^ (0.707 to 1.101) |

Note: *CI*: confidence interval; ®: Reference category; IPTp-SP: Intermittent preventive treatment of malaria in pregnancy with sulfadoxine-pyrimethamine; ANC: Antenatal care; *** *P* < 0.001, ** *P* < 0.01, * *P* < 0.05

^1^Specification 1 included pregnant women who have been pregnant only once. Number of observations (*n* = 6285), Censored observations (*n* = 291), Uncensored observations (*n* = 5994), Wald chi2(14): 51.18, Prob>chi2: *P* < 0.001, and *λ*: -0.88 (*P* < 0.001).

^2^Specification 2 included pregnant women who have been pregnant multiple times. Number of observations (*n* = 23,630), Censored observations (*n* = 1835), Uncensored observations (*n* = 21,795), Wald chi2(15): 184.93, Prob>chi2: *P* < 0.001, and *λ*: -0.70 (*P* < 0.001).

**Table S2.** Determinants of the number of IPTp-SP in Senegal, as per Heckman selection model estimates (robustness analysis results)

|  | **(1)^1^** | **(2)^2^** |
| --- | --- | --- |
| **Outcome Equation for number of IPTp-SP (95% *CI*)** | | |
| **Readiness** | 0.310^***^ (0.212 to 0.407) | 0.709^***^ (0.541 to 0.877) |
| **Place of residence** |  |  |
| Urban ^®^ | 1 | 1 |
| Rural | -0.003 (-0.036 to 0.030) | 0.434^***^ (0.280 to 0.588) |
| **Readiness*Place of residence** |  |  |
| Readiness*Urban ^®^ | – | 1 |
| Readiness*Rural | – | -0.586^***^ (-0.789 to -0.383) |
| **Education level** |  |  |
| No education ^®^ | 1 | 1 |
| Primary | 0.087^***^ (0.055 to 0.119) | 0.089^***^ (0.057 to 0.121) |
| Secondary and higher | 0.157^***^ (0.118 to 0.196) | 0.156^***^ (0.117 to 0.195) |
| **Religion** |  |  |
| Muslim ^®^ | 1 | 1 |
| Others | -0.009 (-0.087 to 0.068) | -0.004 (-0.081 to 0.074) |
| **Age^3^ (years)** |  |  |
| 20–24 ^®^ | 1 | 1 |
| 25–29 | 0.026 (-0.008 to 0.060) | 0.026 (-0.008 to 0.061) |
| 30–34 | 0.091^***^ (0.055 to 0.127) | 0.091^***^ (0.055 to 0.127) |
| 35–39 | 0.118^***^ (0.078 to 0.158) | 0.119^***^ (0.079 to 0.159) |
| **Wealth index quintile** |  |  |
| Poorest ^®^ | 1 | 1 |
| Poorer | 0.009 (-0.026 to 0.044) | 0.009 (-0.026 to 0.044) |
| Middle | 0.006 (-0.033 to 0.044) | 0.007 (-0.031 to 0.046) |
| Richer | 0.036 (-0.010 to 0.081) | 0.048^*^ (0.002 to 0.093) |
| Richest | 0.031 (-0.022 to 0.085) | 0.051 (-0.003 to 0.105) |
| **Selection Equation for ANC (95% *CI*)** | | |
| **Readiness** | -0.604^***^ (-0.827 to -0.381) | -0.606^***^ (-0.830 to -0.382) |
| **Religion** |  |  |
| Muslim ^®^ | 1 | 1 |
| Others | 0.178^*^ (0.017 to 0.338) | 0.174^*^ (0.013 to 0.335) |
| **Age^3^ (years)** |  |  |
| 20–24 ^®^ | 1 | 1 |
| 25–29 | -0.097^*^ (-0.173 to -0.022) | -0.098^*^ (-0.174 to -0.022) |
| 30–34 | -0.189^***^ (-0.266 to -0.111) | -0.190^***^ (-0.267 to -0.113) |
| 35–39 | -0.272^***^ (-0.353 to -0.191) | -0.273^***^ (-0.354 to -0.193) |
| **Number of children aged 5 and under in household** | | |
| 0 ^®^ | 1 | 1 |
| 1 | 0.585^***^ (0.463 to 0.707) | 0.586^***^ (0.463 to 0.708) |
| 2–3 | 0.726^***^ (0.609 to 0.844) | 0.727^***^ (0.609 to 0.845) |
| ≥ 4 | 0.756^***^ (0.636 to 0.875) | 0.756^***^ (0.636 to 0.876) |
| **Education level** |  |  |
| No education ^®^ | 1 | 1 |
| Primary | 0.123^***^ (0.054 to 0.191) | 0.123^***^ (0.054 to 0.192) |
| Secondary and higher | 0.161^***^ (0.072 to 0.249) | 0.162^***^ (0.074 to 0.251) |
| **Wealth index quintile** |  |  |
| Poorest ^®^ | 1 | 1 |
| Poorer | 0.216^***^ (0.148 to 0.284) | 0.217^***^ (0.148 to 0.285) |
| Middle | 0.348^***^ (0.271 to 0.426) | 0.349^***^ (0.271 to 0.427) |
| Richer | 0.361^***^ (0.266 to 0.455) | 0.363^***^ (0.268 to 0.459) |
| Richest | 0.327^***^ (0.215 to 0.439) | 0.328^***^ (0.216 to 0.441) |
| **Place of residence** |  |  |
| Urban ^®^ | 1 | 1 |
| Rural | -0.116^**^ (-0.185 to -0.048) | -0.119^**^ (-0.187 to -0.050) |
| **Year** |  |  |
| 2012–2013 ^®^ | 1 | 1 |
| 2014 | 0.558^***^ (0.484 to 0.632) | 0.558^***^ (0.484 to 0.632) |
| 2015 | 0.979^***^ (0.893 to 1.065) | 0.981^***^ (0.895 to 1.067) |
| 2016 | 0.958^***^ (0.875 to 1.040) | 0.963^***^ (0.880 to 1.045) |
| 2017 | 0.982^***^ (0.890 to 1.075) | 0.971^***^ (0.879 to 1.064) |
| 2018 | 0.937^***^ (0.811 to 1.062) | 0.912^***^ (0.786 to 1.037) |
| 2019 | 0.997^***^ (0.784 to 1.209) | 0.964^***^ (0.751 to 1.176) |

Note: *CI*: confidence interval; ®: Reference category; IPTp-SP: Intermittent preventive treatment of malaria in pregnancy with sulfadoxine-pyrimethamine; ANC: Antenatal care; *** *P* < 0.001, ** *P* < 0.01, * *P* < 0.05

^1^Specification 1 used the malaria service readiness as the main explanatory variable, adjusting for women’s characteristics. Number of observations (*n* = 24,370), Censored observations (*n* = 1428), Uncensored observations (*n* = 22,942), Wald chi2(12): 175.86, Prob>chi2: P < 0.001, and *λ*: -0.77 (*P* < 0.001).

^2^Specification 2 added the interaction between urban-rural settings and the malaria service readiness. Number of observations (*n* = 24,370), Censored observations (*n* = 1428), Uncensored observations (*n* = 22.942), Wald chi2(13): 211.30, Prob>chi2: *P* < 0.001, and *λ*: -0.76 (*P* < 0.001).

^3^Samples from both the youngest age group (15 to 19 years) and the oldest age group (40 to 49 years) were excluded.


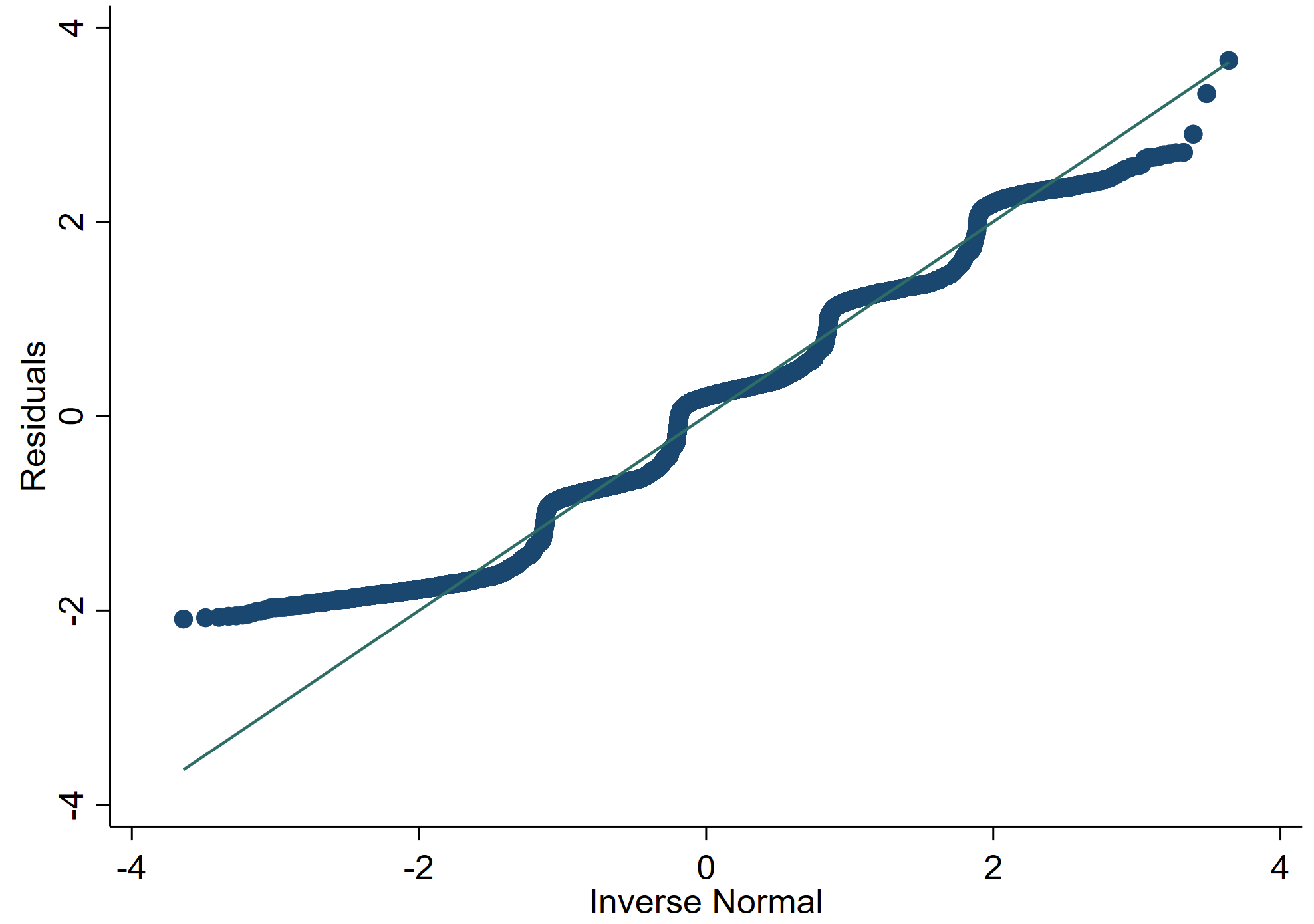


**Figure S1.** Quantile-Quantile Plot of Residuals for Normality Assessment


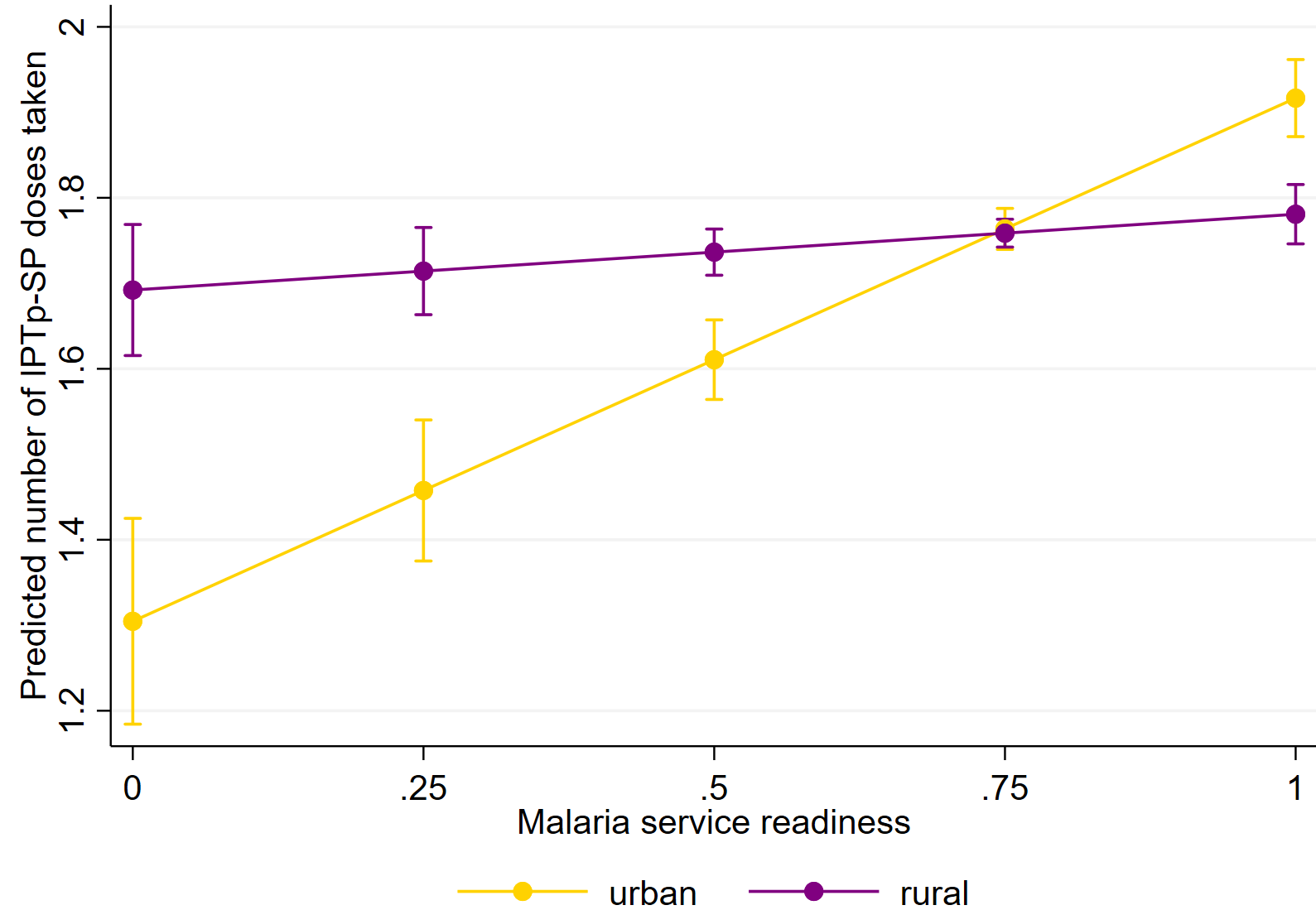


**Figure S2.** Interaction plot of the effects of urban-rural settings and malaria service readiness on IPTp-SP

Note: IPTp-SP: Intermittent preventive treatment of malaria in pregnancy with sulfadoxine-pyrimethamine

Figure S2 shows the effects and 95% confidence intervals of malaria service readiness and urban-rural settings on the number of IPTp-SP received by pregnant women (*n* = 29,915). The x-axis is the malaria service readiness, and the y-axis is the number of IPTp-SP received by pregnant women. The gold color and purple color in the figure represent urban and rural areas, respectively.


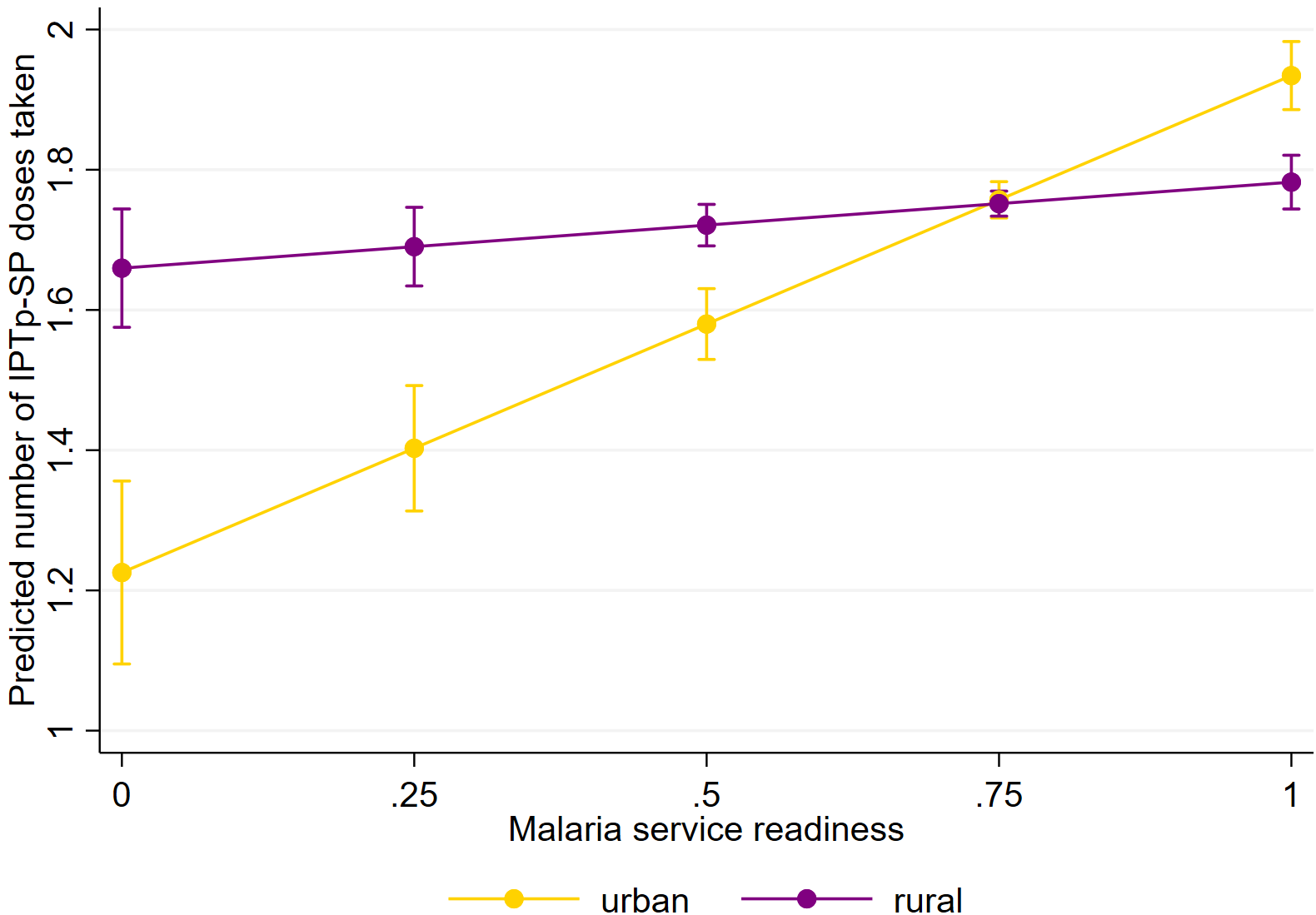


**Figure S3.** Interaction plot of the effects of urban-rural settings and malaria service readiness on IPTp-SP by robustness analysis results

Note: IPTp-SP: Intermittent preventive treatment of malaria in pregnancy with sulfadoxine-pyrimethamine. Figure S3 shows the effects and 95% confidence intervals of malaria service readiness and urban-rural settings on the number of IPTp-SP received by pregnant women aged from 20 to 39 (*n* = 24,370). The x-axis is the malaria service readiness, and the y-axis is the number of IPTp-SP received by pregnant women. The gold color and purple color in the figure represent urban and rural areas, respectively.
